# Regulation of Enhancers by SUMOylation Through TFAP2C Binding and Recruitment of HDAC Complex to the Chromatin

**DOI:** 10.21203/rs.3.rs-4201913/v1

**Published:** 2024-04-02

**Authors:** Tharindumala Abeywardana, Xiwei Wu, Shih-Ting Huang, Grace Aldana Masangkay, Andrei S. Rodin, Sergio Branciamore, Grigoriy Gogoshin, Arthur Li, Li Du, Neranjan Tharuka, Ross Tomaino, Yuan Chen

**Affiliations:** University of California, San Diego; Toni Stephenson Lymphoma Center Beckman Research Institute, City of Hope; University of California, San Diego; Toni Stephenson Lymphoma Center Beckman Research Institute, City of Hope; Toni Stephenson Lymphoma Center Beckman Research Institute, City of Hope; Toni Stephenson Lymphoma Center Beckman Research Institute, City of Hope; Toni Stephenson Lymphoma Center Beckman Research Institute, City of Hope; Toni Stephenson Lymphoma Center Beckman Research Institute, City of Hope; Toni Stephenson Lymphoma Center Beckman Research Institute, City of Hope; University of California, San Diego; Harvard Medical School Taplin Mass Spectrometry Facility; University of California, San Diego

**Keywords:** SUMO, TFAP2C, AP-2, SUMOylation, RNA splicing, spliceosome, H3K27Ac, histone remodeling, enhancers

## Abstract

Enhancers are fundamental to gene regulation. Post-translational modifications by the small ubiquitin-like modifiers (SUMO) modify chromatin regulation enzymes, including histone acetylases and deacetylases. However, it remains unclear whether SUMOylation regulates enhancer marks, acetylation at the 27th lysine residue of the histone H3 protein (H3K27Ac). To investigate whether SUMOylation regulates H3K27Ac, we performed genome-wide ChIP-seq analyses and discovered that knockdown (KD) of the SUMO activating enzyme catalytic subunit UBA2 reduced H3K27Ac at most enhancers. Bioinformatic analysis revealed that TFAP2C-binding sites are enriched in enhancers whose H3K27Ac was reduced by UBA2 KD. ChIP-seq analysis in combination with molecular biological methods showed that TFAP2C binding to enhancers increased upon UBA2 KD or inhibition of SUMOylation by a small molecule SUMOylation inhibitor. However, this is not due to the SUMOylation of TFAP2C itself. Proteomics analysis of TFAP2C interactome on the chromatin identified histone deacetylation (HDAC) and RNA splicing machineries that contain many SUMOylation targets. TFAP2C KD reduced HDAC1 binding to chromatin and increased H3K27Ac marks at enhancer regions, suggesting that TFAP2C is important in recruiting HDAC machinery. Taken together, our findings provide insights into the regulation of enhancer marks by SUMOylation and TFAP2C and suggest that SUMOylation of proteins in the HDAC machinery regulates their recruitments to enhancers.

## INTRODUCTION

Enhancers and super-enhancers (SEs) are chromatin regions regulates gene transcription and thus play critical roles in the regulation of gene expression programs in pluripotency, cell lineage development and in diseases, such as cancer (1–4). Enhancers and SEs are highly enriched for active histone marks H3K27Ac (the acetylation at the 27th lysine residue of the histone H3 protein) and transcription factors. However, it remains unclear how SUMOylation regulates enhancer marks H3K27Ac genome-wide. Small ubiquitin-like modifications (SUMOylation) predominately occur to nuclear proteins including histone acetylases and deacetylases (5–7). SUMOylation is the covalent conjugation of a small ubiquitin-like modifier (SUMO) to target proteins (8). There are at least three SUMO isoforms in mammals, namely SUMO1, SUMO2 and SUMO3. SUMOylation occurs through a cascade of enzymatic activities catalyzed by an E1 activating enzyme that consists of two subunits SAE1 and UBA2 (aka SAE2), an E2-conjugating enzyme and several E3 ligases. Desumoylases, on the other hand, remove SUMO proteins from their targets, making SUMOylation a highly dynamic modification. SUMOylation adds a new docking site for protein-protein interactions through SUMO-interacting motifs (9, 10) or hinders protein-protein interactions by masking an existing binding site. Although many proteins involved in histone deacetylation are substrates of SUMO modification (5–7), the role of SUMOylation in regulating H3K27Ac is not well defined.

TFAP2C, also known as AP-2γ, is a member of the AP-2 family of developmentally regulated transcription factors that consists of five members, AP-2α, AP-2β AP-2γ, APδ and APε that share similar DNA-binding sequences (11). The AP-2 family members form homo- or heterodimers in driving gene expression due to their high sequence similarity (12). TFAP2C is an important regulator of enhancer elements. For example, it was found that TFAP2C plays critical roles in maintaining pluripotency by binding enhancers specific to naïve pluripotent stem cells (13). TFAP2C was also found to regulate OCT4 naive enhancer in human germline formation (14). TFAP2C plays important roles in cancers; it was shown that the loss of TFAP2C induces epithelial-to-mesenchymal transition (15). Overexpression of TFAP2C prevented breast cancer metastasis (16).

In this study, we discovered that knockdown (KD) of the SUMO activating enzyme catalytic subunit UBA2 reduced H3K27Ac at most enhancers. Bioinformatic analysis revealed that TFAP2C-binding sites are specifically enriched in enhancers and SEs whose H3K27Ac was reduced by UBA2 KD. Genome-wide ChIP-seq analysis in combination with molecular biological methods showed that TFAP2C binding to enhancers and SEs increased upon down regulation of UBA2 or inhibition of SUMOylation by a small molecule inhibitor. However, this is not due to the SUMOylation of TFAP2C itself, because UBA2 KD similarly increased wild type (WT) and SUMOylation defective mutant TFAP2C binding to these SEs. We carried out proteomics analysis of TFAP2C interactome on the chromatin. A subset of TFAP2C interacting proteins is involved in histone deacetylation (HDAC) and chromatin modification, and most of these proteins are SUMOylation substrates. TFAP2C KD reduced HDAC1 binding and increased H3K27Ac marks at examined enhancer regions, supporting the interaction of TFAP2C and HDAC machinery identified by proteomics. Taken together, our multi-omics studies described here identify the role of SUMOylation in regulating H3K27Ac mark at enhancers and SEs through TFAP2C.

## EXPERIMENTAL PROCEDURES

### Cell Culture and Small Molecule Inhibition

HCT116 cells stably expressing Tet-On shUBA2 (17, 18) were grown in Dulbecco’s Modification of Eagle’s Medium (DMEM) at 37°C with 5% CO_2_. Media was supplemented with 10% Tet System Approved FBS (Clontech), 100 U/ ml-penicillin, and 100 mg/ml streptomycin. UBA2 knockdown was induced by treating the cells for 5 days with 1 μg/mL of doxycycline as previously shown 17,19). Plasmid transfection was performed with PolyJet^™^ In Vitro DNA Transfection Reagent (SignaGen Laboratories) and siRNA transfection was performed with Lipofectamine RNAiMAX Transfection Reagent (ThermoFisher Scientific) according to the respective manufacturers’ protocols.

### TAK-981 was purchased from MedChemExpress (Cat. # HY-111789).

Whole cell lysate was prepared by lysing the cell in 1X RIPA buffer (25 mM Tris pH 7–8, 150 mM NaCl, 0.1 % SDS, 0.5% sodium deoxycholate, 1 % Triton X-100 or NP-40).

### Site-Directed Mutagenesis

HA-TFAP2C-K10R mutation was performed using QuickChange II XL Site-directed Mutagenesis Kit (Agilent). The following primer set was used for the mutagenesis. TFAP2C K10R-Forward-GGAAAATAACCGATAATGTCAGGTACGAAGAGGACTGCGAG and TFAP2C K10R-Reverse-CT CGCAGTCCT CTT CGTACCT GACATTAT CGGTTATTTT CC.

### Chromatin Immunoprecipitation

Chromatin immunoprecipitation was performed using SimpleChIP^®^ Plus Enzymatic Chromatin IP Kit (Cell Signaling Technology) according to the manufacturer’s protocol. The following antibodies were used for ChIP Assays. anti-HA (CST; #3724S), anti-SUMO1 (CST; #4940), anti-SUMO2–3 (M114–3), anti-H3K27Ac (ab4729), anti-H3K4me3 (Millipore; 04–745), anti-H3K27me3 (ab6002) and anti-Med1(A300–793A). Enrichment analysis at enhancer elements was performed by real-time qPCR using the primers listed in Table S1. For qPCR, a QuantStudio5 real time PCR system and PowerUp SYBR Green Master Mix (Thermo Fisher Scientific) were used. Enrichment was calculated using the comparative Ct method. Each value represents the average value ± standard deviation calculated from triplicate qPCR reactions per one representative experiment.

### ChIP-seq Library Preparation and Quantification and Sequencing

Chip-seq libraries were generated using KAPA Hyper Prep Kit following manufacturer’s protocol. ChIP DNA was subjected to end repair with subsequent 3^’^ adenylation to a create 3’dA overhang suitable for adaptor ligation. Illumina adaptors were ligated to both ends of the DNA and amplified using 11 cycles of PCR with primers specific to the adaptor sequences to generate amplicons of approximately 200–500 bp in size. Libraries were purified using the AxyPrep Mag PCR Clean-up kit (Thermo Fisher Scientific). Each library was quantified using a Qubit fluorometer (Life Technologies) and the size distribution was assessed using the 2100 Bioanalyzer (Agilent Technologies, Santa Clara, USA). The amplified libraries were hybridized to the Illumina single end flow cell and amplified using the cBot (Illumina). Single end reads of 50 nt were generated for each sample. ChIP-Sequencing reads were generated on an Illumina HiSeq2500 machine.

### ChIP-seq Analysis

Sequences were aligned to human genome assembly hg19 using NovaSeq c3.02.07. Only reads aligned to unique genome location were retained for further analysis. Peaks were called using MACS v2 with default settings, using the following options bandwidth = 300 and d = 200. Super enhancers were identified using H3K27Ac and Med1 peaks with ROSE algorithm (http://younglab.wi.mit.edu/super_enhancer_code.html). Subsequent analysis was done using customized R scripts and various Bioconductor packages. Peaks were annotated based on Refseq genes to transcription start site (TSS +/− 500bp), promoter region (TSS +/− 1000bp), gene body (TSS +1000 to transcription end), and intergenic regions. For differential peak identification for TFAP2C, the MACS v2 peaks from multiple samples were merged, and reads falling into each merged region in each sample were counted and scaled to the same total aligned reads. Then log_2_FC were calculated between different sample groups and *p* value was calculated using t-test. Differential peaks were selected with *p* value < = 0.01 and fold change greater than 2. Each targeted region is separated into equal size bins of 100 bp. The reads falling into each bin were counted and scaled by total aligned reads in each sample. These bin counts data were either used to generate a heatmap directly, or the average scaled read counts were calculated and plotted for all the bins in the region. Motif analysis of enhancers was done using the Bioconductor package “ChromeVAR “. Specifically, the enhancer regions are subject to motif matching to the Jaspar motif database, with default sensitivity settings of “ChromeVAR” package (< 0.00005).

### Experimental Design and Statistical Rationale for Proteomics Analysis

For mass spectrometry analysis, HA immunoprecipitation was performed on chromatin-bound (CB) fractions prepared from untransfected, HA-TFAP2C transfected doxycycline untreated (−Dox) and treated (+ Dox) HCT116-Tet-On-ShSAE2 (UBA2) cells. Although there was no replication for each condition, proteins consistently identified from −Dox and + Dox conditions and not from the control untransfected cells are highly reproducible and thus, −Dox and + Dox conditions were used as independent biological replicates for identification of TFAP2C interactome.

Specifically, cells were lysed (30 min, on ice) in 3 volumes of cytoplasmic buffer (10 mM Tris-HCl pH 7.5, 0.34 M sucrose, 3 mM CaCl2, 2 mM MgCl2, 0.1 mM EDTA, 1 mM DTT, 0.5% NP40, 40 mM NEM) containing protease and phosphatase inhibitors. The nuclear pellet was collected by centrifugation (2400 × g, 5 min). Nuclei were then resuspended in 3 volumes of nuclear buffer (20 mM HEPES pH 7.5, 1.5 mM MgCl2, 1 mM EDTA, 150 mM KCl, 0.1% NP40, 1 mM DTT, 10% Glycerol) and homogenized with a 21G1/2 needle. The intact chromatin pellet was collected after centrifugation (18,000 × g, 30 min). To obtain CB fraction, the chromatin pellet was incubated with 2 volumes of nuclease buffer (20mM HEPES pH 7.5, 1.5mM MgCl2, 1 mM EDTA, 150mM KCl, 10% Glycerol, 0.5 U μl - 1 benzonase) overnight at 4°C, and the supernatant was collected as the CB fraction. To immuno-purify HA-tagged protein complexes, chromatin extracts were diluted with 1V of binding buffer (10 mM HEPEs, pH7.5, 10 mM KCl, 0.25 M NaCl, 1.5 mM MgCl2, 1 mM EDTA, 10% Glycerol and 0.5% Triton X-100) and incubated overnight with HA –tag antibody (Cell Signaling Technology (CST), C29F4) at 4°C. The proteins were next incubated with 30 μl of protein G agarose dynabeads (Invitrogen) for 2 h at 4°C. Beads were washed three times with binding buffer and boiled with 2X SDS loading buffer for SDS-PAGE. Bands were excised and submitted for analysis at Taplin Mass Spectrometry Facility at Harvard University.

Excised gel bands were cut into approximately 1 mm3 pieces. Gel pieces were then subjected to a modified in-gel trypsin digestion procedure (19). Gel pieces were washed and dehydrated with acetonitrile for 10 min followed by removal of acetonitrile. Pieces were then completely dried in a speed-vac. Rehydration of the gel pieces was with 50 mM ammonium bicarbonate solution containing 12.5 ng/μl modified sequencing-grade trypsin (Promega, Madison, WI) at 4°C. After 45 min, the excess trypsin solution was removed and replaced with 50 mM ammonium bicarbonate solution to just cover the gel pieces. Samples were then placed in a 37°C room overnight. Peptides were later extracted by removing the ammonium bicarbonate solution, followed by one wash with a solution containing 50% acetonitrile and 1 % formic acid. The extracts were then dried in a speed-vac (~ 1 hr). The samples were then stored at 4°C until analysis.

On the day of analysis, the samples were reconstituted in 5–10 μl of HPLC solvent A (2.5% acetonitrile, 0.1% formic acid). A nano-scale reverse-phase HPLC capillary column was created by packing 2.6 μm C18 spherical silica beads into a fused silica capillary (100 μm inner diameter x ~ 30 cm length) with a flame-drawn tip (20). After equilibrating the column each sample was loaded via a Famos auto sampler (LC Packings, San Francisco CA) onto the column. A gradient was formed, and peptides were eluted with increasing concentrations of solvent B (97.5% acetonitrile, 0.1% formic acid). As peptides were eluted, they were subjected to electrospray ionization and then entered into an LTQ Orbitrap Velos Pro ion-trap mass spectrometer (Thermo Fisher Scientific, Waltham, MA). Peptides were detected, isolated, and fragmented to produce a tandem mass spectrum of specific fragment ions for each peptide.

All raw files were analyzed together in MaxQuant version 2.4.0.0. Derived peaks were searched against the reference human proteome downloaded from Uniport (https://www.uniprot.org/proteomes/UP000005640) and the built-in frequently observed protein contaminant list. Search parameters include semi-specific trypsin digest allowing up to two missed cleavage with no crosslink, carbamidomethylation of cystine and N-acetylation of protein N-termini were set as fixed post-translational modification, oxidation of methionine was set as variable modification. Multiplicity was set to 1, main search peptide tolerance was set to 4.5 ppm, isotope match tolerance was set to 2 ppm and centroid match tolerance was set to 8 ppm. Peptide-spectrum match (PSM) false discovery rate (FDR) and protein FDR were both set to 1%. Razor protein FDR and second peptide feature were both enabled. Label Free Quantification (LFQ) was enabled for relative quantification. The minimum ratio count was set to 1. The normalization type was classic and Fast LFQ with LFQ intensity minimum number of neighbors set to 3 and the maximum number of neighbors set to 6. Parameters not specified were all left as default. The proteinGroups.txt file output from Maxquant was then used as input for downstream analysis in Perseus version 2.0.11 (21) and using SAINTexpress (Significance Analysis of INTeractome) (22) through APOSTL (Automated Processing of SAINT Templated Layouts) (23).

For Perseus, the proteinGroup.txt file was input and formatted as a matrix. Initial filtering steps remove contaminants, decoys, and proteins that were identified with less than two unmodified peptides. The LFQ intensity was logarithmized (log2) and each sample was grouped individually as ctrl, −Dox and + Dox. After grouping, each group was annotated with identifiers such as gene name and protein ID. After the LFQ intensities were logarithmized, the matrix was further filtered to eliminate proteins that were not at least present in 1 of the 3 samples. Missing values were imputed with values representing a normal distribution with a downshift of 1.8 standard deviation and a width of 0.25 standard deviation. The filled-in matrix then allowed downstream analysis including principal component analysis and scatter plot visualization. A separated matrix was derived from this processed matrix with protein that appears in control manually removed from the matrix entries of the −Dox and + Dox protein list.

For data analysis with SAINTExpress, APOSTL, a pipeline that automates the data analysis using SAINTExpress within the open-source Galaxy framework, was also utilized for identifying interacting protein partners of TFAP2C. Analysis workflow was set up based on the default galaxy workflow on Galaxy Server (http://apostl.moffitt.org/). This pipeline generates necessary input files (bait, prey, inter) and pipes them directly into SAINTExpress analysis. In the SAINT pre-processing step, Maxquant output file peptide.txt and Fasta file of the human protein were taken as input. Bait files are generated with the bait create module. Prey and Inter file were automatically generated. The prey file was then used as input for CRAPome search to distinguish commonly identified contaminations of affinity purification from the rest of the identified proteins. SAINTExpress analysis took the prey file, bait file and inter file generated in the previous step as input, with number of replicates set to 1 with virtual control used option set to false. APOSTL interactive analysis took the output SAINT_output file, prey file, craptome file and interfile to generate data QC plot with SAINT score cutoff set to 0.9. SAINT_output file was also used to generate the interaction file with saint score cut off of 0.9 and consensus path database score cut off set to 0.8 for human species. NSAF (Normalized Spectral Abundance Factor) scoring Table, a similar measurement to saint score for the statistical likelihood of identified interaction representing true interaction, was also generated from SAINT_outputfile, Crampome file, prey file and inter file.

### mRNA Extraction, mRNA Sequencing Library Preparation and Sequencing

Total mRNA was extracted from cells using miRNeasy RNA isolation kit (Qiagen) per manufacturer’s protocol. RNA concentration was measured by NanoDrop 1000 (Thermo Fisher Scientific, Waltham Massachusetts, US) and RNA integrity was determined using Bioanalyzer (Agilent). Library construction of 280 ng total RNA for each sample was made using KAPA mRNA HyperPrep Kit (Illumina Platforms) (Kapa Biosystems, Wilmington, USA) according to the manufacturer’s instructions. PCR amplified for 10 cycles to create the final libraries. Libraries were purified using the AxyPrep Mag PCR Clean-up kit (Thermo Fisher Scientific). Each library was quantified using a Qubit Fluorometer (Life Technologies) and the size distribution was assessed using the 2100 Bioanalyzer (Agilent Technologies, Santa Clara, USA). Sequencing was performed on an Illumina^®^ Hiseq 2500 (Illumina, San Diego, CA, USA) instrument using the TruSeq SR Cluster Kit V4-cBot-HS (Illumina^®^) to generate 51 bp single-end reads sequencing with v4 chemistry. Quality control of RNA-Seq reads was performed using FastQC.

### RNA-seq Analysis

The sequences were aligned to human genome assembly hg19 using Tophat2 v2.0.14. For each sample, expression counts for refseq genes were summarized by HTseq, and reads per kilobase of transcript per million mapped reads (RPKM) were calculated. Count normalization and differential expression analysis between groups were conducted using Bioconductor package “edgeR”. Heatmaps were generated using cluster v3.0 and Java Treeview v2. The pathway analysis was performed using GSEA and hallmark pathways in mSigDB.

### Statistical Approach for Experiments Other Than Proteomics

Data shown in bar graphs are mean ± STDEV. P-values were evaluated using Student’s t-test. A *p*-value < 0.05 was considered statistically significant. Stepwise regression was conducted by using the REG procedure from SAS 9.4 with a significance level of F statistics less than 0.05.

## RESULTS

### UBA2 KD Reduces H3K27Ac Marks at Enhancers

**Figure 1:**
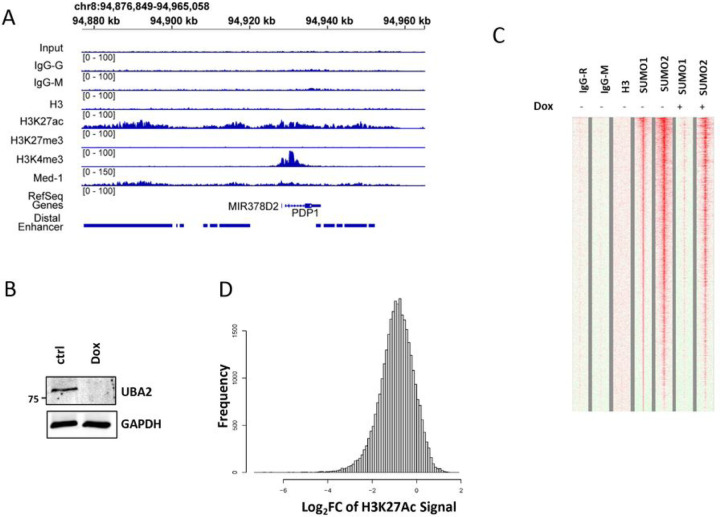
SUMOylation regulates H3K27Ac enhancer marks and TFAP2C binding to enhancers. A) Histone marks at enhancers at a representative locus. B) Western blot analysis of Uba2 protein in whole cell extract. GAPDH was used as a loading control. C) ChIP-seq data of SUMO1 and SUMO2/3 peaks with and without UBA2 KD by adding Dox. D) Enhancer peaks responsive to UBA2 KD and most of these enhancer peaks were down regulated upon UBA2 KD.

SUMOylation regulates many proteins involved in histone modification, including HDAC complexes (24). However, the role of SUMOylation in regulating H3K27Ac enhancer mark genome-wide is not well understood. Therefore, we focused our analysis on enhancers and SEs. We investigated the chromatin landscape of histone marks H3K27Ac, H3K27me3 and H3K4me3 along with Med1 (Mediator Complex Subunit 1) in HCT116 cells by performing genome-wide ChIP-seq analysis. There are previous enhancer and SE analysis performed on the HCT116 cell line that was used to validate our results (3, 25). An example of ChIP-seq peaks of chromatin marks is shown in Fig. 1A. To prevent influence of H3K27Ac peaks from promoters on enhancer analysis, we defined distal enhancers after calling H3K27Ac broad peaks using the MACS2 software (26) and excluded any peaks that are within 2 kilobase (kb) upstream and 1 kb down stream of transcription start site (TSS) of any genes defined in Gencode. This results in 31,719 distal enhancer peaks. Our mapping of distal enhancers and SEs, H3K27me3 and H3K4me3 peaks matches well with that previously reported (3, 25), validating our ChIP-seq experimental and bioinformatic methodology (Supplemental Fig. 1A).

To understand the role of SUMOylation in regulating enhancers, the SUMOylation pathway was suppressed by UBA2 KD using an inducible Tet-On-shUBA2 system (18). Doxycycline (Dox) treatment successfully reduced Uba2 protein levels (Fig. 1B). Additionally, we performed ChIP-seq analysis of SUMO1 and SUMO2 and 3 peaks on the chromatin. UBA2 KD reduced SUMO1 and SUMO2/3 peaks genome-wide as observed previously (27) (Fig. 1C). There was not a strong overlap between the distal enhancer peaks and SUMO1 or SUMO2/3 peaks (Supplemental Fig. 1B). However, we observed down-regulation of 86% of H3K27Ac peaks at enhancers in response to UBA2 KD (Fig. 1D).

### UBA2 KD Increased TFAP2C Binding to Enhancers

**Figure 2:**
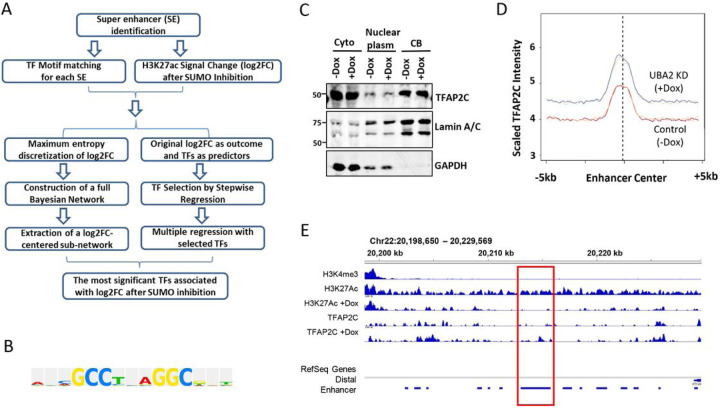
SUMOylation regulates TFAP2C occupancy at enhancers. A) Bioinformatics analysis workflow of the multivariate analysis using BNs and stepwise regression methods to identify candidate DNA-binding proteins including transcription factors (TF). B) TFAP2C binding motif is enriched in the enhancers whose H2K27ac mark is reduced by UBA2 KD. C) Western blot analysis of TFAP2C in sub-cellular fractions (lower panel) in control and UBA2 KD cells. GAPDH and LaminA/C were used as cellular compartment markers and loading controls. “Cyto”, cytoplasmic; CB, chromatin-bound fraction. D) Average ChIP-seq signal for TFAP2C with or without UBA2 KD in a 10 kb window around enhancers. The read coverage at 100 bp bins within the SE center +/−5kb in UBA2KD and control samples were counted and scaled by the total aligned reads. The average coverage across all the enhancers was then calculated and plotted. Light and dark blue lines correspond to biological duplicates with Dox-induced UBA2 KD. Orange and red lines correspond to biological duplicates without Dox-induced UBA2 KD. E) A representative distal enhancer region showing H3K4me3 peaks, H3K27Ac peaks, and TFAP2C peaks with and without Dox-induced UBA2 KD. The region highlighted in red shows H2K27Ac peak reduction and TFAP2C peak increase upon UBA2 KD.

We hypothesized that the reduced H3K27Ac mark upon UBA2 KD is mediated by transcription factors and DNA-binding proteins recruited to enhancer regions. We carried out bioinformatics analysis using two different approaches (Fig. 2A). In one approach, we performed multivariable analysis using Bayesian networks (BNs) and maximum entropy-based discretization as previously described (28). In another approach, we performed feature selection using a stepwise regression. To avoid artificially large fold changes caused by low read coverage, we only retain the peaks with read counts greater or equal to 50 in at least one sample (5,548 peaks). The binding motifs of several members of AP2 families, including TFAP2A, TFAP2B, TFAP2C, and TFAP2E, are significantly enriched among the enhancers down regulated by UBA2 KD (Fig. 2B) (Supplementary Table S2 and S3). This is consistent with the fact that the TFAP2 family members have homologous DNA-binding domains (11).

Next, we aimed to examine how TFAP2C binding to chromatin is modulated by UBA2 KD, and its connection with enhancers. To determine the distribution of TFAP2C on chromatin in HCT116 cells, we adapted a previously established method of performing ChIP-seq analysis of exogenously expressed HA-tagged TFAP2C in cells, using an anti-HA antibody (29), because we could not obtain high quality IP of TFAP2C with commercially available antibodies. Biological duplicates of samples with and without (control) UBA2 KD were analyzed. UBA2 KD did not alter TFAP2C protein expression (Fig. 2C) or localization to cytoplasm (cyto), nuclear plasm or chromatin-bound fractions (CB) (Fig. 2C). Despite the fact that TFAP2C protein level and localization are largely unaffected by UBA2 KD, we observed a significant increase of TFAP2C peaks in the enhancer regions on the chromatin upon UBA2 KD (Fig. 2D and 2E). An example of such regions is shown in Fig. 2E. TFAP2C peaks within each sample were identified using the MACS2 software (26). These results suggest increased TFAP2C binding at enhancers with UBA2 KD.

### TFAP2C’s Own SUMOylation Does Not Regulate Its Binding to Enhancers

**Figure 3:**
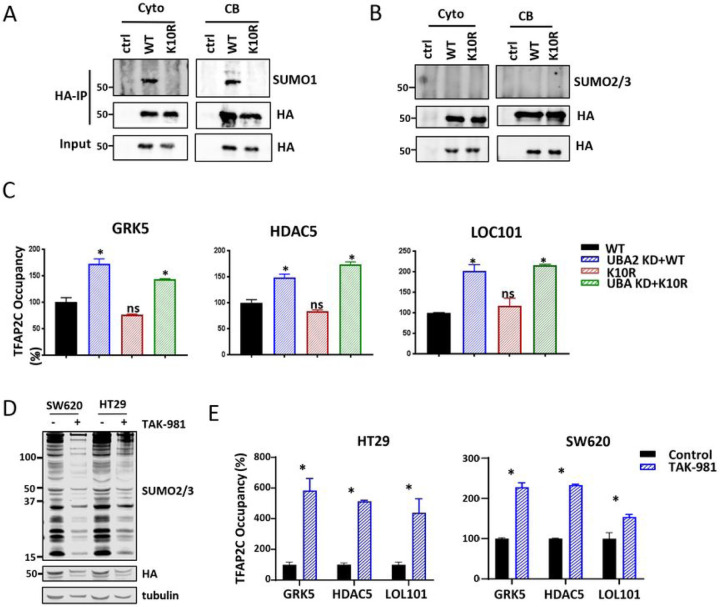
SUMOylation of TFAP2C itself does not affect its chromatin binding. A, B) Western blot analysis of HA-TFAP2C wild-type and SUMOylation deficient mutant K10R modifications by SUMO1 (A) and SUMO2–3 (B) in cytoplasmic (Cyto) and chromatin-bound (CB) fractions. C) ChIP analysis of HA-TFAP2C and HA-TFAP2C K10R occupancy at selected SE regions in HCT116 cells overexpressing HA-TFAP2C with and without UBA2 KD. ChIP signal was initially normalized to 2% input and depicted as fold change of the HA-TFAP2C wildtype ChIP signal in control cells. Each average value ± standard deviation was calculated from triplicate qPCR reactions per one representative experiment out of two independent experiments. *p* values were calculated by t-test analysis for statistically significant differences. *p* values equal to or less than 0.05 is indicated with an asterisk (*). D) SUMOylation inhibition by the SUMO E1 inhibitor TAK-981 (100 nM) was investigated in HT29 and SW260 cells expressing HA-TFAP2C. Global SUMO2 and 3 modifications were detected by western blot with an anti-SUMO2 and 3 antibody. Tubulin was used as a loading control. E) ChIP analysis of HA-TFAP2C occupancy at selected SE regions in HT29 and SW620 cells expressing HA-TFAP2C with and without SUMOylation inhibition by TAK-981. ChIP signal was initially normalized to 2% input and depicted as fold change of the HA-TFAP2C normalized signal in control cells. Each average value ± standard deviation was calculated from triplicate qPCR reactions with one representative experiment out of two shown. *p* values were calculated by t-test analysis for statistically significant differences. *p* values equal to or less than 0.05 are indicated with an asterisk (*).

Next, we investigated how SUMOylation regulates TFAP2C binding to SE and enhancer regions. Previous studies have reported TFAP2C SUMOylation (11, 29). We validated previous findings that TFAP2C is SUMO1 modified at K10 but not SUMO2/3 modified in both cell cytoplasmic and chromatin-bound fractions by conducting HA-TFAP2C immuno-precipitation from cytoplasmic and chromatin fractions, followed by probing with SUMO1 and SUMO2/3 specific antibodies (Fig. 3A and 3B). Then, we interrogated whether SUMOylation of TFAP2C itself regulates its occupancy at several SEs. Towards this end we performed local ChIP analysis at several SE regions (Fig. 3C) with an HA antibody with cells expressing either WT or SUMOylation defective K10R mutant of HA-TFAP2C in control or UBA2 KD cells. SUMOylation deficient K10R mutant was similarly enriched at SEs as WT TFAP2C upon UBA2 KD (Fig. 3C).

We further investigated whether the regulation of TFAP2C binding to enhancer regions is dependent on SUMOylation. We used a highly specific and potent clinical stage small molecule pharmacological SUMOylation inhibitor TAK-981 (30). In addition, we determined whether the effect is limited to HCT116 by examining two additional cell lines SW620 and HT29. TAK-981 inhibited global SUMOylation in SW620 and HT29 cell lines (Fig. 3D). Similar to UBA2 KD, TAK-981 increased TFAP2C recruitment to the same SEs in SW620 and HT29 as in HCT116 upon UBA2 KD (Fig. 3E). Taken together, SUMOylation inhibition increases TFAP2C binding to enhancer regions and this effect is cell line independent. In addition, SUMO-dependent TFAP2C binding on chromatin is not regulated by TFAP2C’s own SUMOylation, but likely is due to TFAP2C’s interactome.

### Identification of TFAP2C Interactome on The Chromatin

**Figure 4:**
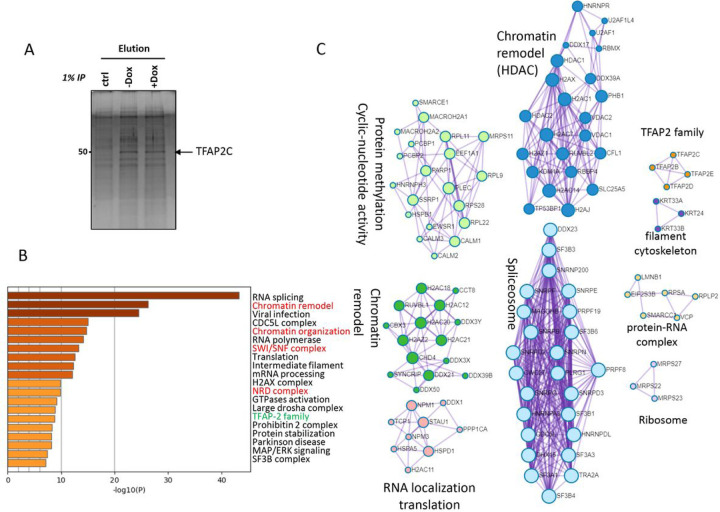
Profiling TFAP2C interacting proteins by proteomics approach. A) HA-TFAP2C was purified from the chromatin bound fractions of the HA-TFAP2C expressing control and UBA2 knockdown HCT116 cells using anti-HA agarose beads. HA-immunoprecipitation from un-transfected cells served as the negative control to eliminate non-specific proteins. Proteins were eluted and analyzed by SDS-PAGE. The gel was silver stained. The arrow indicates the HA-TFAP2C bands. Proteins were analyzed by mass spectrometry. B) Functional enrichment of TFAP2C interactome from analysis using Metascape (31). C) The PPI networks of TFAP2C interactome from analysis using Metascape. The color of the circles refers to molecular complex detection (MCODE) clusters and are labeled accordingly.

We investigated the TFAP2C interactome on chromatin, which has not been previously described. We performed mass spectrometry analysis of HA-tagged TFAP2C purified from the chromatin-bound fractions of WT and UBA2 KD HCT116 cells by immunoprecipitation (IP) using an HA-tagged antibody under the condition that also co-IP TFAP2C-associated proteins or protein complexes (Fig. 4A). Proteomic analysis was also conducted with IP samples using the same procedure and reagents from cells that did not express HA-tagged TFAP2C to eliminate non-specific proteins. Each lane (Fig. 4A) was cut into bands and submitted for proteomic analysis.

Raw data files of the three different conditions were analyzed together using MaxQuant version 2.4.0.0, and the peaks were used to search the reference human proteome and the built-in frequently observed protein contaminant list. Carbamidomethylation of cystine and acetylation of protein N-termini were set as post-translational modifications, and oxidation of methionine was set as variable modification. Both PSM (peptide spectrum match) FDR and protein identification FDR were set to 1%. Label Free Quantification (LFQ) was enabled for relative quantification. The proteinGroups.txt file output from Maxquant was then used as input for downstream analysis using Perseus version 2.0.11 (21) and in SAINTexpress (22) through APOSTL (23).

TFAP2C was only identified from both co-IP samples and not in the control sample (Supplemental Fig. 2A and B), confirming the co-IP and protein identification methods. Although there were no replications for each condition, proteins identified from co-IP under −Dox and + Dox conditions and not from the control are highly reproducible (Supplemental Fig. 2C, Table S4). Thus, −Dox and + Dox conditions can be used as independent biological replicates for identification of TFAP2C interactome.

The TFAP2C interactome on the chromatin was used for analysis of functional enrichment (Fig. 4B) and protein-protein interaction (PPI) networks (Fig. 4C) using Metascape (31). One functional pathway identified is the regulation of TFAP2 family proteins (Fig. 4B) and the identification of other TFAP2 (AP-2) family of proteins in the TFAP2C interactome (Fig. 4C). This is consistent with previous findings that TFAP2 family members form heterodimers with one another in driving gene expression due to their high sequence similarity (12), providing support of the approach for identification of the TFAP2C interactome.

The top functions of the TFAP2C interactome are RNA splicing and chromatin modifications (Fig. 4B). Consistently, one major complex includes the spliceosome as shown by (Fig. 4C). Except HNRNPA3 associated with the A complex and DDX23 associated with the B complex of the spliceosome, seven proteins are associated with both A and B complexes of the spliceosome including SF3B3, SF3B6, SNRPB, SNRPG, SNRPD3, SF3B1, and DHX15 (32). Eight proteins in the TFAP2C interactome are associated with the B and C complex, including SNRNP200, SNRPF, SNRPE, PRPF19, CWC27, PLRG1, PRPF8, and CDC5L (32). Finally, SNRPD2, SF3A3, SF3A1, SF3B4 are associated with more than two complexes of the spliceosome (32). Many of these proteins are SUMOylation substrates (24).

The TFAP2C interactome is enriched in chromatin remodeling and histone deacetylation (colored in red in Fig. 4B). Consistently, two main PPI networks are associated with chromatin remodeling (Fig. 4C) (31). Both histone deacetylases HDAC1 and HDAC2 are found in one PPI network of TFAP2C interactome (Fig. 4C) (33). Many proteins in both PPI networks are known SUMOylation substrates (24). Because we observed changes of H3K27Ac marks upon UBA2 KD (Fig. 1D), we focused our studies of the TFAP2C interactome related to HDAC.

### TFAP2C Directly Participates in The Recruitment of HDAC1 to Enhancers

**Figure 5:**
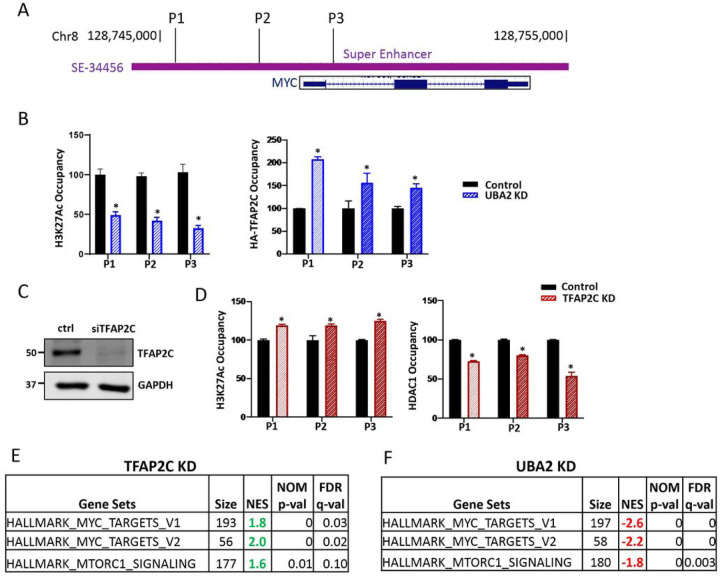
TFAP2C participates in recruiting HDAC complexes. A) Location of the MYC SE (SE-34456, purple) and MYC coding sequence (blue) as shown by UCSC Genome Browser. The positions of the three probes, P1, P2 and P3, used for ChIP-PCR are indicated. B) ChIP analysis of H3K27Ac enrichment at MYC super enhancer with and without UBA2 KD. C) Western blot analysis showing efficient TFAP2C KD following 48 hrs of siRNA targeting TFAP2C coding region. GAPDH was used as a loading control. D) H3K27Ac (left panel) and HDAC1 (right panel) enrichment at MYC super enhancer with and without UBA2 KD. ChIP signal was initially normalized to 2% input and depicted as fold change of the ChIP signal in control cells. Each average value ± standard deviation was calculated from triplicate qPCR reactions with one representative experiment out of three independent experiments shown. *p* values were calculated by t-test analysis for statistically significant differences. *p* values equal to or less than 0.05 is indicated with an asterisk (*). E) GSEA analysis mRNA-seq data obtained from control and TFAP2C knockdown cells. F) GSEA analysis mRNA-seq data obtained from control and SAE2 knockdown cells.

Because it was shown that TFAP2C regulates MYC (34, 35), we used the well-established enhancer of the Myc gene (SE-34456, Fig. 5A) for validation studies. We performed H3K27Ac ChIP in control and UBA2 KD cells and examined the MYC SE using three probes (P1 centered at chr8:128,745,443, P2 centered at chr8:128,747,286, and P3 centered at chr8:128,749,112). UBA2 KD resulted in reduced H3K27Ac at these regions of the MYC SE (Fig. 5B, left panel). Thus, the MYC SE is negatively regulated by UBA2 KD like many other enhancer regions (Fig. 1D). Local ChIP-PCR demonstrated that UBA2 KD increased TFAP2C enrichment at this locus (Fig. 5B, right panel), as we observed in other enhancer regions (Fig. 2D, Fig. 3C, Fig. 3E).

Next, we investigated whether TFAP2C plays a critical role in deacetylation at the MYC SE. Towards this end, we performed local ChIP-PCR at MYC SE in control and TFAP2C KD cells (Fig. 5C). Our data indicated that TFAP2C KD enhanced H3K27Ac signal at the MYC SE (Fig. 5D, left panel). We probed the occupancy of HDAC1 at the MYC SE by performing local ChIP-PCR. TFAP2C KD reduced HDAC1 localization to the MYC SE (Fig. 5D, right panel), corresponding to enhanced H3K27Ac (Fig. 5D, left panel). These results support the critical role of TFAP2C in recruiting HDAC1 to the MYC SE, providing support for the proteomics finding (Fig. 4C).

To further examine the regulation of MYC enhancer using genome-wide data, we performed genome-wide mRNA-seq analysis on RNA extracted from control and TFAP2C KD cells. TFAP2C KD increased Myc-dependent gene expression as shown by Gene Set Enrichment Analysis (GSEA) (Fig. 5E). These effects are opposite to UBA2 KD that increase TFAP2C binding to the chromatin; GSEA of mRNA seq data from control and UBA2 KD cells showed a significant reduction in enrichment for Myc-dependent gene expression with UBA2 KD (Fig. 5F). Together, these results support the finding that SUMOylation inhibition enhances TFAP2C binding and deacetylation of selective enhancers to regulate gene expression.

## DISCUSSION

In this study, we show that SUMOylation regulates enhancer marks. To rule out potential off-target effects of shRNA, we validated our findings using a highly specific and potent clinical stage SUMOylation inhibitor (30). In addition, to rule out the role of SUMOylation in regulating H3K27Ac marks in promoter regions that was previously investigated (36), we specifically analyzed distal enhancers and inhibiting SUMOylation reduces H3K27Ac in most distal enhancers. This effect is linked to enhanced TFAP2C binding to the enhancer regions upon SUMOylation inhibition. We have shown that TFAP2C binding to enhancers was not affected by its own SUMOylation, but likely regulated by SUMOylation of TFAP2C interacting proteins.

TFAP2C has been found to regulate enhancer elements in gene expression programs maintaining pluripotency (13, 37), germline formation (14) and oncogenesis (15); however, TFAP2C’s interactome on the chromatin has not been reported previously. Therefore, we carried out a proteomics analysis of TFAP2C interactome on the chromatin by proteomics. The TFAP2C interactome on the chromatin with and without UBA2 KD are similar, with similar functional enrichment and PPI interaction networks (Fig. 4, Supplemental Fig. 2 and Table S4). A validation of our proteomic approach is the identification of other TFAP2 family members in the TFAP2C interactome. This is consistent with previous findings that TFAP2 family members form heterodimers with one another in driving gene expression (12). Among the TFAP2C interacting proteins that are reproducibly identified from two conditions, with and without UBA2 KD, are histone deacetylases HDAC1 and HDAC2 and their interacting proteins (Fig. 4C). The association between TFAP2C and HDAC is validated by our analysis using the MYC SE and Myc-driven gene expression programs (Fig. 5). Knockdown of TFAP2C reduced HDAC1 binding to the MYC enhancer and increased the H3K27Ac mark, suggesting that TFAP2C is critically involved in increasing HDAC binding to this enhancer. This finding is consistent with previous report that TFAP2C binding to the MYC SE suppresses MYC expression (34). The association of TFAP2C with RNA slicing complexes has not been previously reported and requires future investigations.

The link between SUMOylation and TFAP2C uncovered in this study provides an understanding of the reported opposing functions of TFAP2C and SUMOylation. Loss of TFAP2C induces epithelial-to-mesenchymal transition (EMT) (15) and overexpression of TFAP2C prevented breast cancer metastasis (16). In contrast, high expression of SUMOylation enzymes correlates to poor prognosis in cancer patients (17, 18, 38, 39). Besides cancer, both TFAP2C and SUMOylation were found to play important roles in maintaining pluripotent stem cell state. TFAP2C promotes reprogramming of somatic cells to iPSCs (35), while inhibition of SUMOylation promotes cell reprogramming to pluripotent state (40, 41).

One limitation of our study is that we did not define a specific mechanism that regulates TFAP2C binding to the chromatin in a SUMOylation-dependent manner. It is possible that SUMOylation of a protein (or proteins) in the HDAC complex hinders interaction with TFAP2C; thus, inhibition of SUMOylation promotes TFAP2C interactions with HDAC complexes that enhances TFAP2C binding to the chromatin due to HDAC’s own chromatin-binding activity. Because most proteins identified in the TFAP2C interacting HDAC PPI network are SUMOylation substrates, it would take considerable effort to identify the specific SUMOylated protein in this regulation and thus beyond the scope of the current study. None-the-less, our studies suggest a potential role of SUMOylation of HDAC PPI interacting network proteins in controlling their recruitment to enhancers.

**Figure 6:**
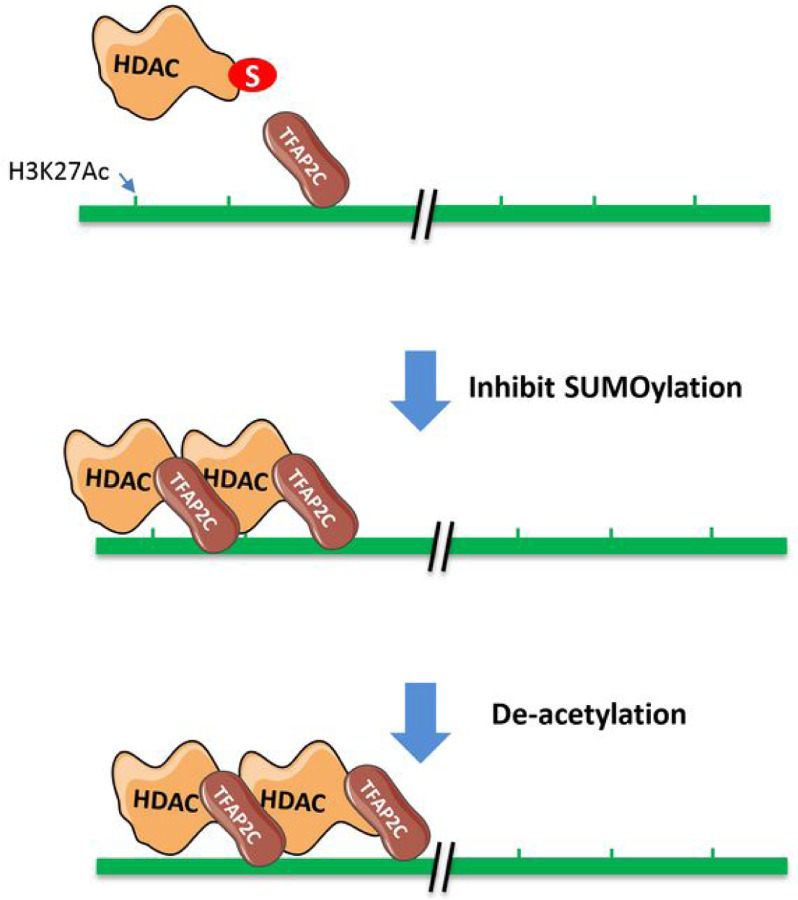
A schematic illustration summarizing our data regarding how SUMOylation regulates H3K27ac in distal enhancers.

In summary, we discovered that SUMOylation regulates H3K27Ac marks of many distal enhancers and SEs, at least in part, through TFAP2C recruiting HDAC complexes to enhancers (Fig. 6). Because of heterodimerization of TFAP2 (AP-2) family of proteins (12), our findings may have implications for other AP-2 members.

## Data Availability

ChIP-seq data and RNA-seq data have been deposited in the GEO database with accession number GSE171817. The mass spectrometry proteomics data have been deposited to the ProteomeXchange Consortium via the PRIDE (42) partner repository with the dataset identifier PXD045644.
